# Internal electric field boosting visible photocatalytic degradation of antibiotics by flower-like CeO_2_/Bi_2_S_3_ S-scheme heterojunctions†

**DOI:** 10.1039/d5ra02077h

**Published:** 2025-06-23

**Authors:** Shanlin He, Yawei Du, Chen Li, Claudia Li, Jingde Li, Jaka Sunarso, Sibudjing Kawi, Yinhui Li

**Affiliations:** a School of Chemical Engineering and Technology, Hebei University of Technology Tianjin 300400 P. R. China liyinhui@hebut.edu.cn; b Chemical Materials Technology Laboratory, China National Offshore Oil Corporation Tianjin Chemical Research & Design Institute Tianjin 300131 PR China; c Department of Chemical and Biomolecular Engineering, National University of Singapore 4 Engineering Drive 4 117585 Singapore; d Research Centre for Sustainable Technologies, Faculty of Engineering, Computing and Science, Swinburne University of Technology Jalan Simpang Tiga 93350 Kuching Sarawak Malaysia

## Abstract

An internal electric field can be formed by constructing a heterojunction to achieve effective separation of photogenerated electrons and holes, which is able to solve the problem of easy compounding of photogenerated carriers in a single semiconductor photocatalyst. This research employs a hydrothermal synthesis technique to develop S-scheme heterojunction photocatalysts composed of cerium oxide and bismuth sulfide (CeO_2_/Bi_2_S_3_) and evaluates their efficacy in degrading TC under visible light. The formation of S-scheme heterojunctions was confirmed by X-ray photoelectron spectroscopy (XPS) and density functional theory (DFT) calculations showing that electrons migrate, and the internal electric field of the S-scheme heterojunctions achieves the separation of the electron–hole pairs, retaining the redox capacity of useful electrons and holes, which is responsible for the enhancement of the photocatalytic activity. The synthesized CeO_2_/Bi_2_S_3_-2 photocatalysts demonstrated a TC degradation rate of 82.43% after a duration of 120 minutes under visible light irradiation. The rate constant of this performance was two times greater than that of CeO_2_ alone and 2.75 times greater than that of Bi_2_S_3_. In addition, free radical trapping experiments and electron paramagnetic resonance results confirmed that ·O_2_^−^ and h^+^ are active substances in the photocatalytic reaction process. Liquid chromatography-mass spectrometry (LC-MS) detected possible intermediates and suggested degradation pathways. This study has significant implications for the future development and enhancement of S-scheme heterojunction photocatalysts, contributing to advancements in photocatalytic materials.

## Abstract 2

CeO_2_/Bi_2_S_3_ S-scheme heterojunctions prepared by combining wide bandgap transition metal oxides with narrow-band system semiconductor metal sulfides were used for photocatalytic degradation of tetracycline.

## Graphical abstract

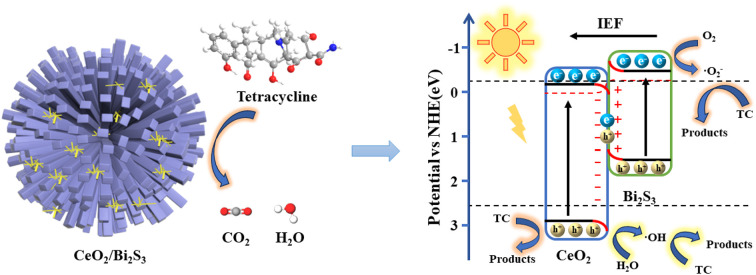

## 1 Introduction

The discovery of antibiotics and their application in disease treatment, alongside the misuse of antibiotics such as tetracycline (TC), have resulted in significant environmental contamination.^1,2^ TC is present as a persistent organic pollutant in the environment, leading to the gradual accumulation and the potential development of microbial resistance, which poses risks to both ecological systems and human health.^3,4^ Consequently, the remediation of TC residues is critical, which might be achieved using various methods such as physical adsorption, biodegradation, and chemical techniques.^5^ Physical adsorption effectively removes contaminants but necessitates frequent replacement of the adsorbent material. Biodegradation employs microorganisms to degrade TC, offering economic advantages and effective performance; however, it often produces by-products that may be more toxic than TC, rendering it a less attractive choice.^6,7^ Chemical methods, such as photocatalytic degradation and precipitation, are also employed. Photocatalytic technology is an advanced oxidation process capable of completely degrading organic pollutants by generating reductive electrons and oxidative holes without producing secondary pollution, garnering significant attention in recent years.^8^

Cerium dioxide (CeO_2_), a widely-used n-type semiconductor, has significant applications in photocatalysis, fuel cells, and industrial catalysis, given its cost-effectiveness, high stability, and low environmental impact.^9,10^ However, its relatively broad energy bandgap of approximately 3.2 eV hinders its ability to absorb visible light effectively. As a result, the electrons generated upon photoexcitation are unable to move to the conduction band (CB) and recombine with holes, severely limiting its photocatalytic efficiency.^11,12^ Recent studies have addressed these limitations through various strategies, including morphological control,^13,14^ metal doping^15–17^ and the fabrication of heterojunctions.^18–20^ For instance, CeO_2_ nanoparticles with a precisely controlled size of 2.1 nm demonstrated a glyphosate decomposition rate 20 times greater than that of larger 4.8 nm nanoparticles, highlighting the significant effect of particle size on photocatalytic activity.^21^ The photocatalytic degradation of TC was markedly enhanced when using H_2_-reduced Mn-doped CeO_2_ compared to pure CeO_2_, with manganese doping facilitating a hierarchical structure and surface atomic arrangement that modifies the electronic properties of the material.^16^ Furthermore, Cu–CeO_2_/BiOBr Z-type heterojunction exhibited reduced electron–hole recombination, with copper doping lowering the energy bandgap of CeO_2_ and the heterojunction structure expanding the visible light absorption range, achieving a 92.3% degradation of sulfathiazole within 90 min.^15^ The construction of heterojunctions has emerged as an effective strategy to enhance photocatalytic performance, with common frameworks including type-II heterojunctions^22^ and Z-type heterojunctions.^23,24^ However, research indicates that traditional type-II heterojunctions face challenges for effective charge separation, while Z-type heterojunctions encounter issues such as redox pair interactions that reduce the available photogenerated electrons and holes, along with a charge transfer mechanism that undermines their purported benefits.^25^

To address these challenges, the step-structured heterojunction (S-scheme) has been proposed,^26^ which integrates an oxidized photocatalyst (OP) with a reduced photocatalyst (RP). This combination creates a catalytic system with enhanced performance, where the CB of the RP is elevated, along with its Fermi level (*E*_f_), both of which are superior to those of the OP, establishing a significant driving force for electron transfer.^27,28^ Upon contact between the RP and OP, an internal electric field (IEF) is generated, facilitating electron migration and promoting the photocatalytic reaction. This IEF enables the recombination of excess electrons in the CB of the OP with the unwanted holes found in the valence band potential (VB) of the RP. This recombination serves to mitigate the losses associated with excess energy, reducing energy losses.^29,30^ Simultaneously, this mechanism ensures the preservation of beneficial electrons and holes, allowing them to maintain their active involvement in the essential redox reactions, thereby enhancing the efficiency of the photocatalytic system. Therefore, there is a need to find a suitable RP semiconductor to construct an S-scheme heterojunction with CeO_2_.

Bi_2_S_3_ functions as an n-type semiconductor characterized by a narrow bandgap, which exhibiting significant light absorption within the visible spectrum and allows for tunability in its bandgap energy.^31,32^ The Bi_2_S_3_ heterojunction, when combined with wide bandgap semiconductors, enhances effective electron transfer. In this heterojunction, electrons migrate from Bi_2_S_3_, which has a comparatively lower CB to a wide bandgap semiconductor with a higher CB. This mechanism not only improves electron mobility but also facilitates the separation of electron–hole pairs generated by light. Such a migration mechanism suggests enhanced visible light absorption when light strikes the heterojunction, ultimately leading to improved efficiency in the separation of photogenerated carriers.^33^ Hydrothermal synthesis of g-C_3_N_4_/Bi_2_S_3_ resulted in 95.6% degradation of Reactive Black 5 and 97.5% degradation of indigo cochineal after 120 min of light exposure.^34^ This research underscores the feasibility of the approach and hints that both CeO_2_ and Bi_2_S_3_ can be used to form highly efficient S-scheme heterojunctions.

In this work, CeO_2_/Bi_2_S_3_ S-scheme heterojunction photocatalysts were synthesized by combining the wide bandgap semiconductor CeO_2_ with the narrow bandgap semiconductor Bi_2_S_3_ using hydrothermal methods, and were used to degrade TC under light conditions. The assessment of the photodegradation efficiency of the CeO_2_/Bi_2_S_3_ S-scheme heterojunction photocatalysts facilitated an initial exploration of the mechanisms and pathways involved in TC degradation, informed by free radical trapping experiments, energy band structure analysis, and intermediate product characterization. The results showed that the CeO_2_/Bi_2_S_3_ S-scheme heterojunction photocatalyst not only enhanced the charge migration efficiency, but also effectively promoted the directional separation of the photogenerated electron–hole pairs through the establishment of an internal electric field, which significantly enhanced the photocatalytic degradation performance while maintaining the strong redox ability of the dominant carriers. The main text of the article should appear here with headings as appropriate.

## 2 Experimental section

### 2.1 Materials

Cerium nitrate (Ce(NO_3_)_3_·6H_2_O), bismuth nitrate (Bi(NO_3_)_3_·5H_2_O), l-ascorbic acid (LA), ethylene glycol (C_2_H_6_O_2_, EG), tetracycline (C_22_H_24_N_2_O_8_, TC) and thiourea (CH_4_N_2_S) were supplied by Shanghai Macklin Reagent Co. Isopropyl alcohol (IPA), sodium hydroxide (NaOH), and sodium ethylenediaminetetraacetic acid (EDTA-2Na) were purchased from Tianjin Kemel Chemical Reagent Co. The water used in the experiment was deionized water. All chemical reagents are directly used without further purification.

### 2.2 Preparation of CeO_2_

60.00 g NaOH in 87.5 mL of water was heated and stirred at 70 °C for 30 min to accelerate the dissolution, and 5.42 g Ce(NO_3_)_3_·6H_2_O in 12.5 mL of deionised water was stirred until it was completely dissolved, and then, the two solutions were mixed slowly and stirred magnetically for 30 min, and the mixture was transferred to 100 mL of a polytetrafluoroethylene-lined stainless steel autoclave, hydrothermal treatment at 100 °C for 24 h, and cooled to room temperature. The mixture was transferred to 100 mL PTFE-lined stainless steel autoclave, hydrothermally treated at 100 °C for 24 h, cooled to room temperature, and the resultant was centrifuged (9000 rpm, 5 min) and washed with water several times until the pH was 7. After drying at 100 °C for 4 h, the product was heated to 500 °C at an elevated rate of 5 °C min^−1^ and calcined in air for 5 h. The CeO_2_ nanorods were obtained by grinding into powder.

### 2.3 Preparation of CeO_2_/Bi_2_S_3_

**Scheme 1:**
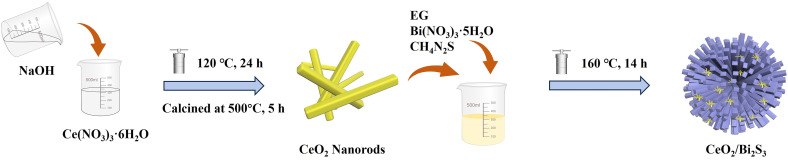
Schematic illustration of the synthesis of CeO_2_/Bi_2_S_3_ photocatalysts.

A total of 0.97 g of Bi(NO_3_)_3_·5H_2_O was combined with 25 mL of EG and stirred for 30 min, resulting in what is referred to as solution A. Solution B was also prepared by incorporating 0.34 g of CeO_2_ and 0.23 g of CH_4_N_2_S was also stirred in 25 mL of EG. Once solution B was successfully prepared, it was gradually combined with solution A and stirred for 30 min to guarantee proper mixing of the two solutions. The ultimate product is subsequently moved to a stainless steel autoclave that has a lining made of polytetrafluoroethylene, and subjected to hydrothermal treatment at 160 °C for 14 h. The resultant products were washed multiple times with water through centrifugation (9000 rpm for 5 min) and then dried at 80 °C for 12 h in a vacuum oven. The CeO_2_/Bi_2_S_3_ composites were ground into powders, and the specific procedure of the experiment is shown in Scheme 1. Based on the different applied molar ratios of CeO_2_ to Bi_2_S_3_ in the composite photocatalysts were of 3 : 1, 2 : 1, 1 : 1 and 1 : 2, the obtained photocatalysts were named CeO_2_/Bi_2_S_3_-1, CeO_2_/Bi_2_S_3_-2, CeO_2_/Bi_2_S_3_-3, and CeO_2_/Bi_2_S_3_-4, respectively. The Bi_2_S_3_ photocatalysts were also prepared without doping CeO_2_, *via* same procedure as described above.

### 2.4 Characterization

A D/max-2500PC (Rigaku) instrument was used to perform powder X-ray diffraction to evaluate the physical phases and crystal structure of the photocatalysts. Concurrently, the morphology of the photocatalysts was assessed using a cold field emission scanning electron microscope (SEM, Hitachi S-4800), enabling the observation of the samples' morphological features at the microscopic scale. Additionally, a high-resolution transmission electron microscope (TEM, JEM-2100F), complemented by an energy dispersive spectrometer (EDS), was utilized to provide further insights into the internal structures and compositions of the photocatalysts. Moreover, to thoroughly explore the chemical composition of the samples and their respective oxidation states, an X-ray photoelectron spectrometer (XPS, Shimadzu AXIS Supra^+^) was employed to characterize the oxidation states. The photocatalysts' light absorption characteristics were evaluated using a UV-Vis Diffuse Reflectance Spectrometer (UV-Vis DRS, V-750 (JASCO)), which provide a quantitative information of their light absorption capabilities. Finally, the investigation of the photogenerated carrier complexation in the photocatalysts was carried out using photoluminescence spectroscopy (PL), with a fluorescence photometer (FLS-1000) excited at 365 nm. Determination of degraded TC intermediates by liquid chromatography-mass spectrometry (Bruker ESI-Q-TOF).

### 2.5 Photoelectrochemical analysis

The transient photocurrent response and electrochemical impedance were assessed with a conventional three-electrode setup utilizing the CHI660E electrochemical workstation. The counter electrode was represented by a Pt electrode, whereas the reference electrode was Ag|AgCl (3 M KCl). A mixture of 10 mg of photocatalysts, 800 μL of ethanol, and 200 μL of Nafion was sonicated for 10 min and applied to a 1 × 1 cm^2^ FTO glass, which was dried and then the FTO glass electrode was clamped to a copper-violet electrode holder as the working electrode. It was submerged in 0.2 M Na_2_SO_4_ solution and placed under the radiation of a 300 W xenon lamp.

### 2.6 Evaluation of photocatalytic performance

The photocatalytic degradation of TC was examined using a 300 W xenon light source (CEL-PF300-T6) equipped with a 420 nm filter to simulate solar light irradiation. A 100 mL solution of TC, with an initial concentration *C*_0_ = 10 mg L^−1^ was transferred into a 200 mL jacketed beaker, into which 20 mg of photocatalyst was introduced. The temperature of the beaker was maintained at 25 °C throughout the experiment using a circulating cooling water system, and the solution was homogenized *via* magnetic stirring. Adsorption equilibrium was established by stirring the solution in the dark for 40 min. Following this, the light source was activated for the photoreaction. After every 20-min intervals, approximately 4 mL of the solution was abstracted with a syringe and filtered through a 0.45 μm water filter to remove the photocatalysts. The reactive radicals produced during photocatalysis were investigated using electron paramagnetic resonance (EPR, Bruker A300, USA). The TC concentration was measured with a UV-visible spectrophotometer (YOKE T3202S) at a designated wavelength of 357 nm, and the degradation rate of TC was assessed in accordance with Beer–Lambert law by utilizing the absorbance measured at that wavelength. The degradation rate of TC (*D*) is computed using eqn (1).1
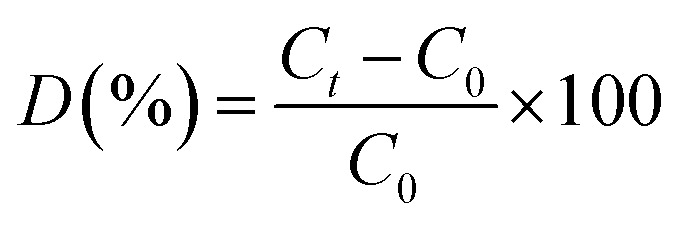
where *C*_*t*_ denotes the concentration at time *t*, while *C*_0_ signifies the initial concentration.

### 2.7 Theoretical calculations

The Vienna *Ab initio* Simulation Package (VASP) was employed to perform all the density functional theory (DFT) calculations employing the projector-augmented wave method (PAW).^35,36^ To enhance the precision and dependability of our calculations, we utilized the Perdew–Burke–Ernzerhof (PBE) exchange–correlation function.^37^ This combination effectively represents electron behavior and associated forces, providing a solid theoretical foundation for our research. Additionally, we employed a plane-wave basis set with a kinetic energy cut-off of 400 eV to ensure the adequacy of our computational approach. We adopted the lattice parameters *a* = 11.771 Å, *b* = 11.324 Å and *c* = 32.670 Å for the CeO_2_/Bi_2_S_3_ heterostructure, which consists of a single layer of Bi_2_S_3_ on the CeO_2_ (111) substrate. In this research, a vacuum layer thickness of 20 Å is established. To accurately characterize the energetic behavior of the system, a cut-off energy of 520 eV was employed for the plane-wave basis set. Brillouin zone integration was conducted using Monkhorst–Pack *k*-point sampling.^38^ Throughout the self-consistent calculations, a convergence energy threshold of 10^−5^ eV was maintained, minimizing the potential influence of numerical instability on the results. Additionally, the maximum allowable stress on each atom was constrained to 0.05 eV Å^−1^.

## 3 Results and discussion

### 3.1 Structural and morphological analysis

**Fig. 1:**
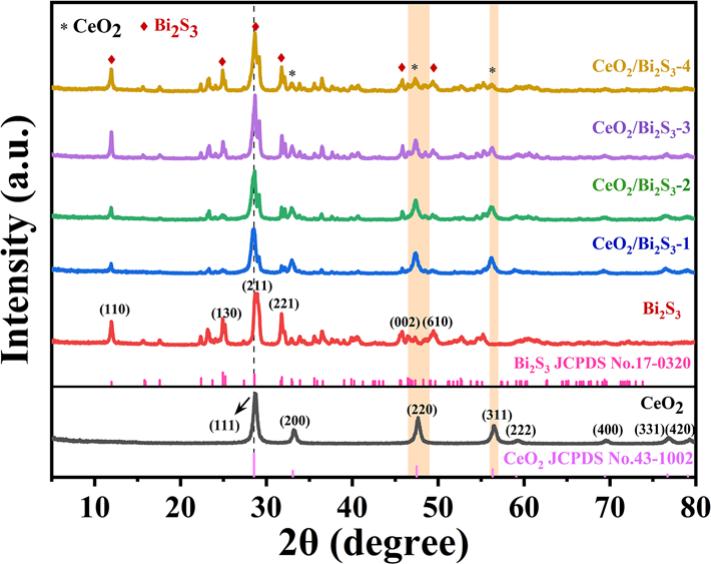
Powder XRD diffraction patterns of CeO_2_, Bi_2_S_3_, and CeO_2_/Bi_2_S_3_ samples.

Fig. 1 presents the powder XRD diffraction patterns for the pure CeO_2_, Bi_2_S_3_ and CeO_2_/Bi_2_S_3_ photocatalysts at varying molar ratios. The distinctive diffraction peaks of the synthesized CeO_2_ (No. 43-1002) appear at 2*θ* = 28.72°, 33.16°, 47.62°, 56.40°, 59.34°, 69.64°, 76.94° and 79.26°, which correlate with the (111), (200), (220), (311), (222), (400), (331) and (420) crystal planes. The absence of impurity peaks indicates successful synthesis of pure CeO_2_. The characteristic diffraction peaks of the as-prepared Bi_2_S_3_ located at 2*θ* = 11.94°, 24.92°, 28.96°, 31.76°, 45.82°, and 49.36° correspond to the peaks of Bi_2_S_3_ (No. 17-0320) at the (110), (130), (211), (221), (002), and (610) crystal faces. The powder XRD patterns of the CeO_2_/Bi_2_S_3_ photocatalysts displayed distinctive peaks corresponding to both CeO_2_ and Bi_2_S_3_. As the ratio of CeO_2_/Bi_2_S_3_ in the material decreases, the diffraction peaks matching the (200), (220), and (311) crystal planes gradually decrease, while the diffraction peaks at the (110), (130), (221), and (610) crystal planes gradually enhance. The shift of the diffraction peaks to a lower angle is due to the doping of Bi into the crystal structure of CeO_2_, which leads to lattice expansion and the shift of the diffraction peaks to a lower angle.^17,39,40^ These observations confirm the successful fabrication of the CeO_2_/Bi_2_S_3_ photocatalysts.

**Fig. 2:**
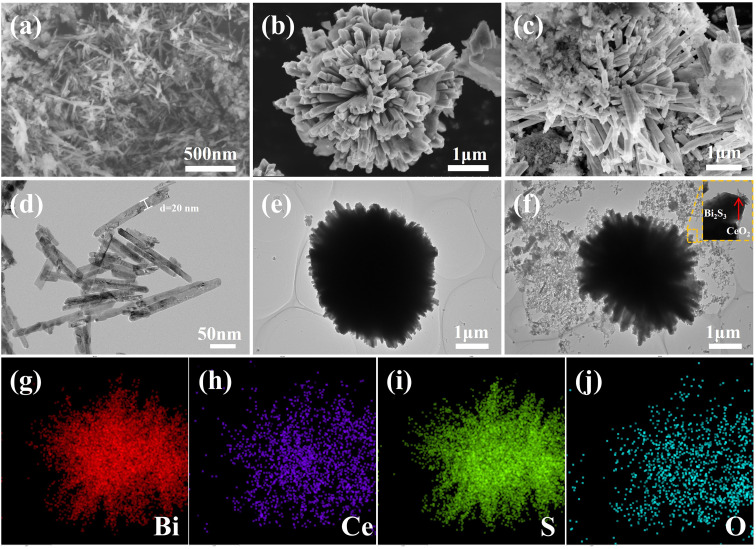
SEM images of (a) CeO_2_; (b) Bi_2_S_3_; (c) CeO_2_/Bi_2_S_3_-2, TEM images of (d) CeO_2_; (e) Bi_2_S_3_; (f) CeO_2_/Bi_2_S_3_-2; EDS elemental distribution mapping of (g) Bi; (h) Ce; (i) S; (j) O in CeO_2_/Bi_2_S_3_-2.

The morphology of the photocatalysts was examined using SEM and TEM. The pure form of CeO_2_ manifests as nanorods averaging 200 and 500 nm in length, as illustrated in Fig. 2(a) and (d). In contrast, Fig. 2(b) and (e) reveal that Bi_2_S_3_ displays a flower-like microarchitecture, roughly 4 μm in size. Fig. 2(c) and (f) demonstrate the doping of nanorods on the surface of the flower-like Bi_2_S_3_, with high-resolution TEM images confirming the strong binding of CeO_2_ and Bi_2_S_3_, indicating successful construction of the heterogeneous junction. Elemental distribution mapping *via* EDS for the CeO_2_/Bi_2_S_3_-2 photocatalyst is illustrated in Fig. 2(g)–(j). The result reveals a uniform distribution of Bi, Ce, S, and O on the surface of CeO_2_/Bi_2_S_3_-2, supporting the effective formation of the heterojunction as well as the successful complexation of CeO_2_/Bi_2_S_3_.

### 3.2 XPS analysis

**Fig. 3:**
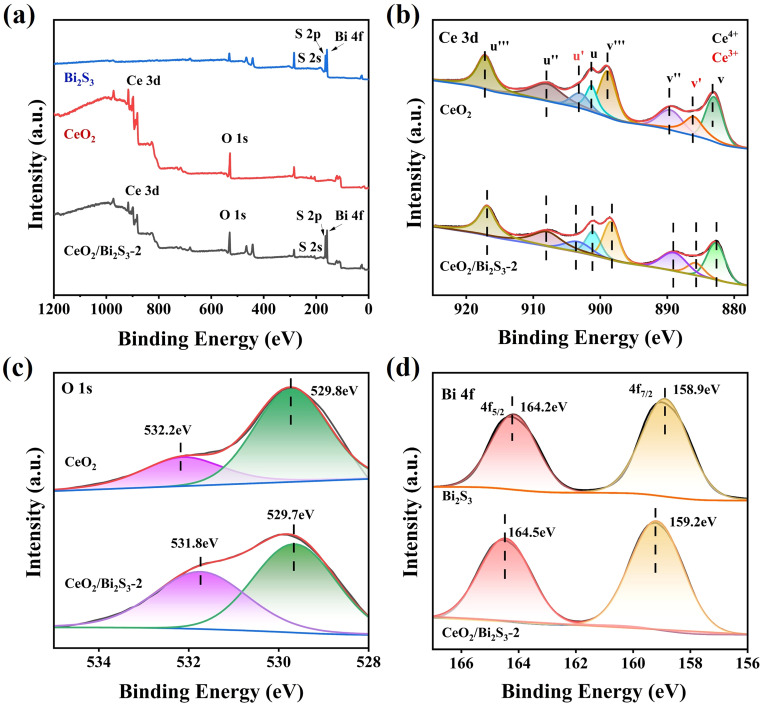
XPS spectra of CeO_2_, Bi_2_S_3_, and CeO_2_/Bi_2_S_3_-2: (a) survey scan spectra; (b) Ce 3d; (c) O 1s; (d) Bi 4f.

The elemental composition and oxidation states of the photocatalysts were analyzed using XPS. As shown in Fig. 3(a), the elemental peaks for both CeO_2_ and Bi_2_S_3_ are present in CeO_2_/Bi_2_S_3_-2, confirming the successful synthesis of the heterojunction. The Ce 3d spectrum, depicted in Fig. 3(b), exhibits both Ce^4+^ and Ce^3+^ components, with eight characteristic peaks, detailed in Table S1.† In this context, the variables *u* corresponds to the spin orbitals of Ce 3d_3/2_ and *v* corresponds to the spin orbitals of Ce 3d_5/2_, while *u*′ and *v*′ pertain to Ce^3+^. Conversely, the variables *u*′′′, *u*′′, *u*, *v*′′′, *v*′′, and *v* are associated with Ce^4+^. The binding energy of the Ce 3d electron in CeO_2_ is higher than that observed in CeO_2_/Bi_2_S_3_-2 photocatalysts. The O 1s of CeO_2_ in Fig. 3(c) has two strong peaks at 529.8 eV and 532.2 eV, corresponding to the lattice oxygen and surface hydroxyl oxygen of CeO_2_, respectively.^41,42^ In contrast, a decrease in the binding energies of Ce 3d and O 1s occurs in CeO_2_/Bi_2_S_3_-2. The Bi 4f spectrum of Bi_2_S_3_ (Fig. 3(d)) has two strong peaks at 158.9 eV and 164.2 eV belonging to Bi 4f_7/2_ and Bi 4f_5/2_, which suggests that the Bi exists in the form of Bi^3+^.^43^ The binding energy of CeO_2_/Bi_2_S_3_-2 is enhanced relative to the Bi 4f spectrum of Bi_2_S_3_. When electrons are lost or gained, the binding energy of the constituent elements increases or decreases accordingly.^29^ This indicates that electron transfer occurs between the contact surfaces of CeO_2_ and Bi_2_S_3_, where the binding energy of Ce and O decreases and electrons are gained, the binding energy of Bi increases and electrons are lost, and electrons are transferred from Bi_2_S_3_ to CeO_2_, leading to the creation of heterojunctions.

### 3.3 Photocatalytic performance test

**Fig. 4:**
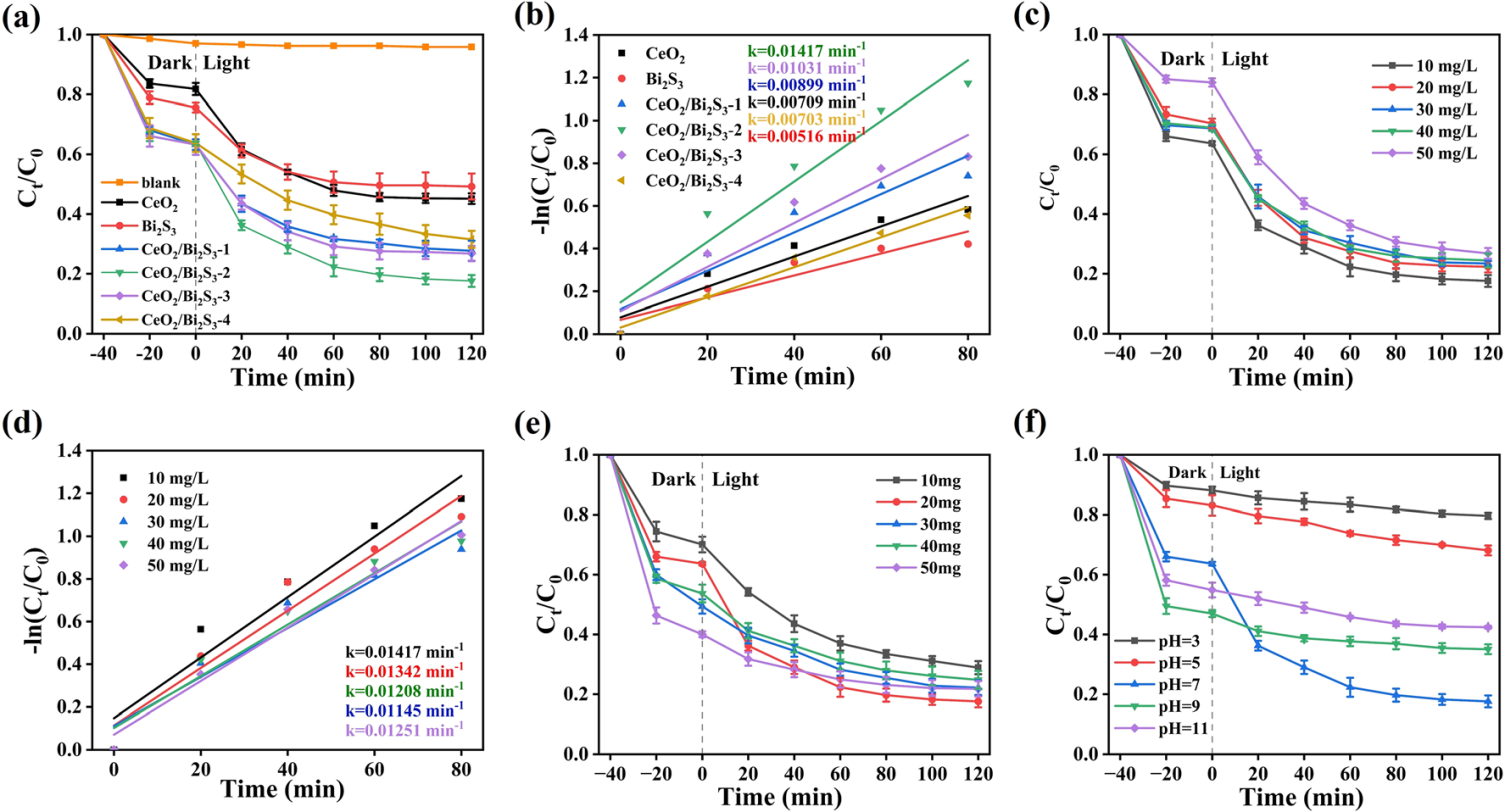
(a) Photocatalytic degradation of TC by as-prepared photocatalysts under simulated solar illumination and (b) its kinetic fitting curves; 20 mg CeO_2_/Bi_2_S_3_-2 degradation of (c) different initial concentrations of TC solution and (d) its kinetic fitting curves; photocatalytic degradation of tetracycline solution by CeO_2_/Bi_2_S_3_-2 at (e) different catalyst dosage (f) different initial pHs.

The prepared photocatalysts effectively degraded TC when exposed to light from a 300 W xenon lamp, and it should be noted that the adsorption of TC molecules by the photocatalysts is not negligible, with adsorption equilibrium reached for all samples after 40 min in the dark. In Fig. 4(a), it is evident that the concentration of TC remained almost the same with no addition of photocatalysts. Pure CeO_2_ and Bi_2_S_3_ achieved photocatalytic degradation rates of 54.85% and 50.78%, respectively, after 120 min. Compared with the pristine materials, the CeO_2_/Bi_2_S_3_ photocatalysts were significantly improved, and the degradation rates of TC by CeO_2_/Bi_2_S_3_-1, CeO_2_/Bi_2_S_3_-2, CeO_2_/Bi_2_S_3_-3 and CeO_2_/Bi_2_S_3_-4 were 72.31%, 82.43%, 73.23% and 68.51% within 120 min, respectively. It is evident that the degradation efficiencies of the CeO_2_/Bi_2_S_3_ photocatalysts initially increased and then decreased with higher Bi_2_S_3_ content, with CeO_2_/Bi_2_S_3_-2 exhibiting the highest performance. Quasi-primary kinetic fitting was performed using eqn (2)

2 −ln(*C*_*t*_/*C*_0_) = *kt*

where *k* represents the apparent reaction rate constant. The kinetic curve indicates that CeO_2_/Bi_2_S_3_-2 exhibits a *k* value of 0.01417 min^−1^, which is higher than all other samples. (Fig. 4(b)). The value observed is significantly greater, demonstrating an increase of two times compared to pure CeO_2_, which has a rate of 0.00709 min^−1^. Additionally, it is 2.75 times higher than the rate for pure Bi_2_S_3_, which is recorded at 0.00516 min^−1^.

To examine how the initial concentration influences the photodegradation of TC, experiments were conducted using varying initial concentrations of TC solutions, with 20 mg of CeO_2_/Bi_2_S_3_-2 employed for each trial. The results are illustrated in Fig. 4(c) and (d). Clearly, an increase in the starting concentration of TC leads to an overall decrease in degradation efficiency, which declines from 82.43% to 73.19%. Additionally, the primary kinetic constant decreased from 0.01417 min^−1^ to 0.01145 min^−1^. At higher initial concentrations, a greater number of TC molecules compete with one another, resulting in fewer molecules being captured and adsorbed by the photocatalysts.^44,45^ In contrast, lower initial concentrations reduce this competitive effect, allowing a greater number of TC molecules to adhere to the photocatalyst's surface.

The quantity of photocatalysts is a significant element that influences the effectiveness of photocatalytic degradation. An investigation was conducted to assess the degradation impact of varying amounts of CeO_2_/Bi_2_S_3_-2 on TC at a concentration of 10 mg L^−1^. Fig. 4(e) reveals that the efficiency of TC degradation increases and subsequently declines as the catalyst amount rises from 10 mg to 50 mg. During the dark reaction phase, an increase in catalyst dosage provides more adsorption sites, thereby enhancing the adsorption capacity. However, the degradation rate of TC exhibits a pattern of initial increase followed by a decrease during the light reaction phase. The peak degradation of TC was observed at an optimized catalyst dosage of 20 mg. This decline in degradation rate at higher catalyst concentrations may be attributed to increased turbidity in the solution, which impairs the catalyst's ability to absorb light, thus reducing the degradation rate of the photoreactive component.^46,47^ Therefore, a dosage of 20 mg is selected as the optimum for subsequent studies.

The pH of the solution is a critical factor influencing the photocatalytic degradation of TC. Variations in pH can significantly alter the interactions between the photocatalyst and the pollutant throughout the reaction process. Consequently, it is essential to optimize the solution's pH by selecting appropriate concentrations of acids and bases, such as 0.1 M HCl and NaOH. In Fig. 4(f), the degradation rates of TC at pH of 3, 5, 7, 9 and 11, the final degradation rate of TC was recorded at 20.31%, 31.86%, 82.43%, 64.96% and 57.60%, respectively. The degradation rate increases as the pH rises from 3 to 7 but declines when the pH exceeds 7. At lower pH levels, ·O_2_^−^ reacts with H^+^ to form H_2_O_2_ and ·O_2_^−^ is consumed in large quantities, resulting in a much lower degradation rate.^48^ At pH 7, photogenerated electrons react more readily with dissolved oxygen to form ·O_2_^−^, resulting in easier separation of carriers, higher electron transfer efficiency and enhanced photocatalytic degradation rates.^49^ In alkaline conditions, the adsorption of TC by the catalyst increases, and the excess TC adsorption blocks the visible light from reaching the catalyst surface, which reduces the light absorption rate of the catalyst, which in turn leads to a lower degradation rate.^50^ Furthermore, in alkaline conditions, h^+^ reacts with OH^−^ to form ·OH, and the h^+^ content decreases, resulting in a lower rate of TC degradation.^51^ In the subsequent free radical trapping experiments, it was demonstrated that the contribution of h^+^ to TC degradation was greater than that of ·OH. Therefore, the degradation rate of TC was reduced under alkaline conditions. The performance of photocatalytic degradation of TC by CeO_2_/Bi_2_S_3_-2 photocatalytic material was compared with previous studies, and as shown in Table 1, the degradation performance of the prepared CeO_2_/Bi_2_S_3_-2 photocatalytic material was superior to that of most of the other reported photocatalytic materials.

**Table 1:** Comparison of TC degradation rate of CeO_2_/Bi_2_S_3_-2 with other photocatalysts

Sample	TC (mg L^−1^)	Irradiation time (minutes)	Degradation (%)	Ref.
CeO_2_/Bi_2_S_3_-2	10	120	82.43	This work
CeO_2_/Bi_2_O_2_CO_3_	20	90	79.50	12
β-Bi_2_O_3_@CeO_2_	10	180	100	19
CeO_2_/CNNS	10	120	79.6	42
Bi_2_O_2_CO_3_/Ti_3_C_2_	20	120	81.00	54
g-C_3_N_4_/CeO_2_	10	160	77.95	55
Bi_2_WO_6_	30	180	79.68	56
2% Au/CeO_2_	20	90	86.40	57
CeO_2_/Co_3_O_4_	20	30	85.35	58
CeO_2_/Co_3_O_4_	20	60	90	59
PDIs/C, N, S-CeO_2_	20	30	80.10	60

### 3.4 Photoelectrochemical analysis

**Fig. 5:**
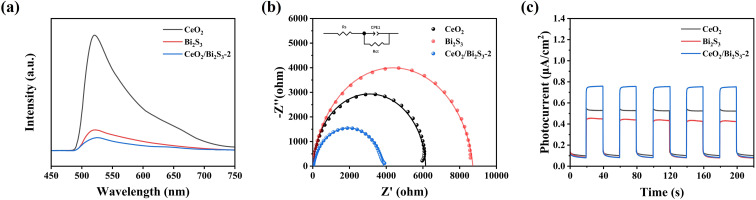
(a) PL, (b) EIS Nyquist plot, and (c) transient photocurrent response of CeO_2_, Bi_2_S_3_ and CeO_2_/Bi_2_S_3_-2.

This research examined the effectiveness of separating photogenerated carriers through photoluminescence spectroscopy. The PL spectra for CeO_2_, Bi_2_S_3_, and CeO_2_/Bi_2_S_3_-2 were recorded with an excitation wavelength of 368 nm. An inverse relationship was identified between PL intensity and the efficiency of photogenerated electron–hole pair separation; specifically, lower PL intensity correlates with a reduced likelihood of electron–hole complex formation, thereby enhancing separation.^52^ The PL intensity of the CeO_2_/Bi_2_S_3_-2 photocatalyst, as shown in Fig. 5(a), is significantly lower when compared to that of both pure CeO_2_ and pure Bi_2_S_3_, indicating effective suppression of electron–hole complexation. This suppression may be attributed to the development of an IEF within the heterojunction that promotes electron–hole pair separation. The electrochemical impedance spectrum (EIS) presented in Fig. 5(b) shows that a smaller curve radius indicates a reduced level of electrochemical impedance.^53^ The impedance curve of CeO_2_/Bi_2_S_3_-2 exhibits a smaller radius when compared to the curves of the pure CeO_2_ and the pure Bi_2_S_3_, suggesting that the heterojunction formed by the composite materials facilitates charge transfer by lowering resistance. Furthermore, findings from the transient photocurrent response of all samples exposed to visible light (Fig. 5(c)) corroborate the EIS results. This evidence underscores the significant advantages of heterojunctions over individual CeO_2_ and Bi_2_S_3_ in terms of the effectiveness of photogenerated carrier separation, thereby enhancing photocatalytic performance.

### 3.5 Stability and cyclic testing

**Fig. 6:**
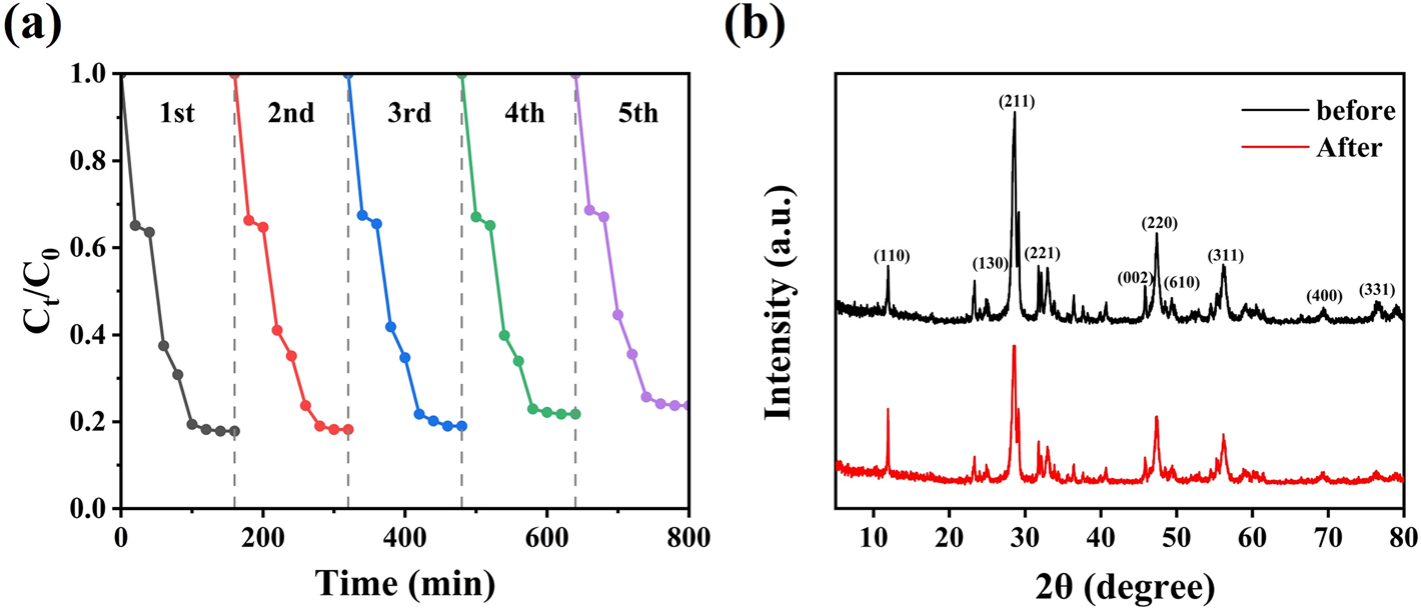
Powder XRD spectra of CeO_2_/Bi_2_S_3_-2 catalyst for (a) 5 cycles of TC degradation experiment and (b) before and after the reaction.

The stability of the CeO_2_/Bi_2_S_3_-2 photocatalysts was assessed through cycling experiments. In these experiments, the CeO_2_/Bi_2_S_3_-2 photocatalysts were collected by filtration after each degradation test, after repeated washing with anhydrous ethanol, and subsequently evaporated excess water in an oven for 12 h at 60 °C before being subjected to cyclic operation again under identical conditions. As depicted in Fig. 6(a), following five cycles of photocatalytic degradation, the rate of photocatalytic degradation dropped from 82.43% to 76.26%, representing a reduction of 6.17%. This decrease is attributed to material loss during the recycling process. Additionally, powder XRD patterns before and after the use of the photocatalyst (Fig. 6(b)) indicated that the structure of CeO_2_/Bi_2_S_3_-2 remained largely unchanged throughout the reaction process, demonstrating the excellent stability and recyclability of the CeO_2_/Bi_2_S_3_-2 photocatalyst.

### 3.6 Photocatalytic mechanism analysis

**Fig. 7:**
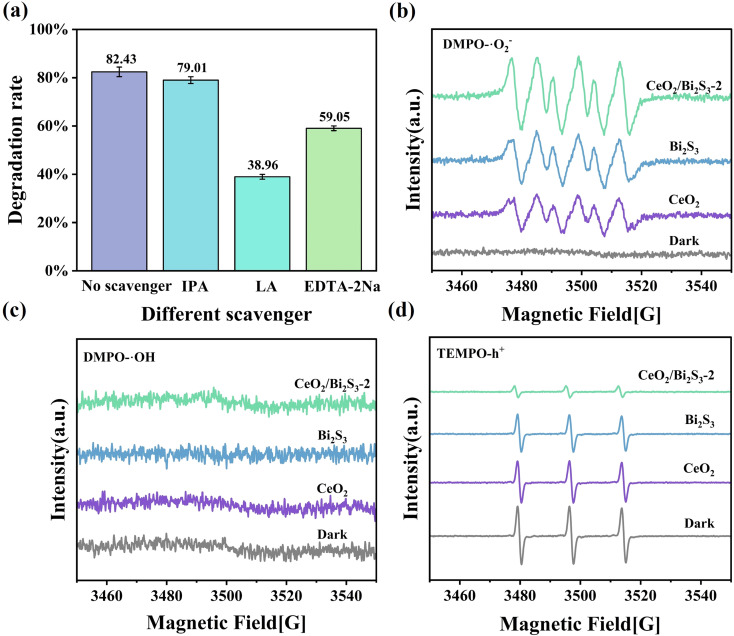
(a) Effect of different scavengers on the photocatalytic degradation of TC by CeO_2_/Bi_2_S_3_-2; EPR signals of (b) DMPO–·O_2_^−^; (c) TEMPO–h^+^; (d) DMPO–·OH.

Experiments were conducted to capture and identify free radicals generated during the process as a way to investigate the fundamental mechanisms responsible for the photocatalytic degradation of TC when utilizing CeO_2_/Bi_2_S_3_-2. EDTA-2Na, LA,^61^ and IPA were utilized as scavengers for various reactive species, specifically h^+^, ·O_2_^−^, and ·OH. The effectiveness of these scavengers in modulating the degradation of TC was evaluated. As depicted in Fig. 7(a), the introduction of IPA resulted into a negligible change in the degradation rate of TC, suggesting that ·OH has a somewhat limited impact on the photodegradation process of TC. Our observations indicated that the inclusion of LA and EDTA-2Na significantly hindered the photocatalytic degradation process of TC. Specifically, we noted a substantial decline in the degradation rate of TC, which plummeted from 82.43% to 38.96% following the addition of LA. Furthermore, the degradation rate was also impacted by the addition of EDTA-2Na, leading to a decrease to 59.05%. These results suggest that the active species ·O_2_^−^ and h^+^ play a primary role in the degradation process, while ·OH has a minimal impact. To further confirm whether ·O_2_^−^, ·OH, and h^+^ are indeed present in the photocatalytic process, we performed a qualitative detection of free radicals using EPR. The ·O_2_^−^ and ·OH radicals were detected using the reagent 5,5-dimethyl-1-pyrroline *N*-oxide (DMPO), and the presence of h^+^ was detected by 2,2,6,6-tetramethylpiperidin-1-oxyl (TEMPO). As shown in Fig. 7(b), no signal of DMPO–·O_2_^−^ was detected under dark conditions, while a significant DMPO–·O_2_^−^ signal was detected after light exposure, which proved that ·O_2_^−^ was produced during the photocatalytic process. The order of DMPO–·O_2_^−^ signal intensity is CeO_2_ < Bi_2_S_3_ < CeO_2_/Bi_2_S_3_-2, which indicates that CeO_2_/Bi_2_S_3_-2 generates more ·O_2_^−^.Whereas ·OH had no obvious signal under both dark and light conditions (Fig. 7(c)), indicating that no ·OH was produced during the whole reaction. From Fig. 7(d), it can be seen that the signal peak of TEMPO decreases in intensity after light exposure, which is due to the reaction between h^+^ and TEMPO, where h^+^ increases and TEMPO consumed by h^+^ increases, resulting in a weakening of the TEMPO signal.^46^ This proves that h^+^ does exist. The intensity of the signal peak for TEMPO–h^+^ was weakest for CeO_2_/Bi_2_S_3_-2, which generated more h^+^. The above results demonstrate that CeO_2_/Bi_2_S_3_ S-scheme heterojunction generates more ·O_2_^−^ and h^+^ radicals involved in the degradation of TC.

**Fig. 8:**
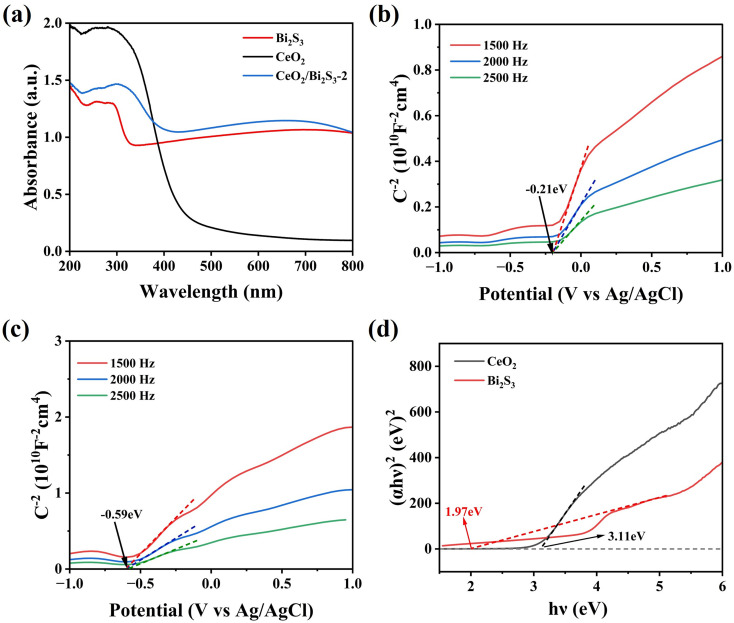
(a) DRS spectra. Mott–Schottky spectra of (b) CeO_2_ and (c) Bi_2_S_3_. (d) Tauc plot of CeO_2_ and Bi_2_S_3_.

To provide additional insight into the mechanism by which CeO_2_/Bi_2_S_3_-2 degrades TC, we analysed the light absorption characteristics of the samples that we had prepared using UV-Vis DRS. The findings presented in Fig. 8(a) indicate that the pure CeO_2_ demonstrates significant light absorption capabilities in the ultraviolet spectrum, whereas it shows minimal absorption in the visible region. Additionally, pure Bi_2_S_3_ shows a robust ability to absorb light within the visible region. The formation of a heterojunction that combines CeO_2_ with Bi_2_S_3_ has led to significant improvements in light absorption properties for CeO_2_/Bi_2_S_3_-2. When comparing its performance to that of pure CeO_2_ and pure Bi_2_S_3_, CeO_2_/Bi_2_S_3_-2 exhibits superior capability to absorb light within the visible spectrum, attributable to the light-absorbing of Bi_2_S_3_ within the heterojunction. The flat band potentials (*E*_fb_) of Bi_2_S_3_ and CeO_2_ were assessed using the Mott–Schottky test. The slopes of the Mott–Schottky curves for both are positive, which proves that both CeO_2_ and Bi_2_S_3_ are n-type semiconductors.^39,44^ The results in Fig. 8(b) and (c) reveal that the *E*_fb_ values for CeO_2_ and Bi_2_S_3_ were −0.21 eV and −0.59 eV, respectively, when measured against the Ag/AgCl reference electrode at a pH 7. The energy levels of *E*_fb_ for both CeO_2_ and Bi_2_S_3_ were evaluated from eqn (3) to be −0.01 eV for CeO_2_ and −0.39 eV (*vs.* NHE) for Bi_2_S_3_.^62^

3 *E*_NHE_ = *E*_Ag/AgCl_ + 0.197

The *E*_fb_ of n-type semiconductors is near the *E*_f_,^63^ and is situated about 0.1 V more positive than the CB.^64,65^ Consequently, the CB levels of CeO_2_ and Bi_2_S_3_ are measured to be −0.11 eV and −0.49 eV (*vs.* NHE), respectively. The bandwidth of the sample is calculated from eqn (4).

4 (*αhv*)^*n*^ = *A*(*hv* − *E*_g_)

5 *E*_VB_ = *E*_CB_ + *E*_g_

where the absorption coefficient, Planck's constant, optical frequency and absorbance are represented by *α*, *h*, *ν*, and *A*, respectively. As direct bandgap semiconductors, CeO_2_ and Bi_2_S_3_ have an *n* value of 2. From Fig. 8(d), the forbidden bandwidths for CeO_2_ and Bi_2_S_3_ are 3.11 eV and 1.97 eV, respectively. Additionally, the VB for CeO_2_ and Bi_2_S_3_ are identified as 2.99 eV and 1.48 eV, respectively, as indicated by eqn (5) (*vs.* NHE).

**Fig. 9:**
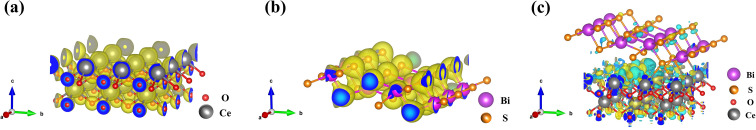
Charge distribution of (a) CeO_2_ and (b) Bi_2_S_3_ before contacting (c) difference in charge density of CeO_2_/Bi_2_S_3_.

The difference in charge density of photocatalysts was calculated using DFT. Fig. 9(a) and (c) show the electron cloud densities of CeO_2_ and Bi_2_S_3_ before contact, respectively. Fig. 9(c) reveals the charge density difference of CeO_2_/Bi_2_S_3_. The yellow areas indicate regions of electron accumulation, while the green areas signify electron depletion. It is clear that electrons accumulated on the CeO_2_ surface, while the Bi_2_S_3_ surface exhibited charge loss. This charge loss is due to the migration of electrons from Bi_2_S_3_ to CeO_2_ following contact, indicating the formation of an IEF between the two surfaces, with the electric field directed from Bi_2_S_3_ towards CeO_2_.

**Fig. 10:**
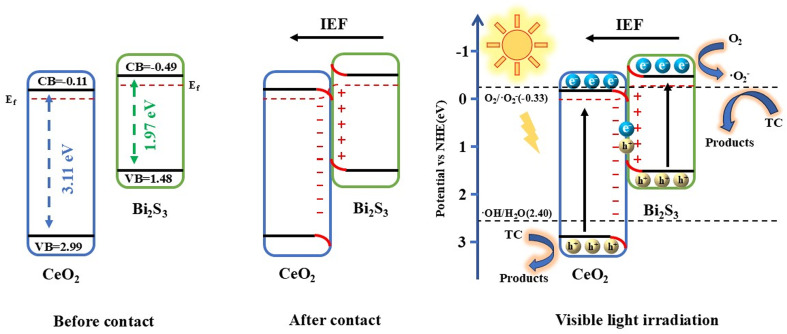
Schematic energy band structure and photocatalytic mechanism of CeO_2_/Bi_2_S_3_-2 photocatalysts.

Based on the experimental results presented in this study, we propose the energy band configurations and charge transfer mechanisms for CeO_2_ and Bi_2_S_3_, both before and after their interaction, as well as the processes related to the photocatalytic breakdown of TC (Fig. 10). Prior to contact, the energy bands of CeO_2_ and Bi_2_S_3_ are interleaved, and the reduced semiconductor Bi_2_S_3_ has a higher *E*_f_ than the oxidized semiconductor CeO_2_. Thus, when the two materials come into contact, the variation in Fermi levels causes the natural movement of electrons from Bi_2_S_3_ to CeO_2_. This process continues until the Fermi levels equalize, resulting in the creation of an IEF at the interface directed from Bi_2_S_3_ towards CeO_2_. Simultaneously, the energy band at the Bi_2_S_3_ interface loses electrons, causing it to curve upwards, while the CeO_2_ interface acquires electrons, which leads to a downward bend. This modification hinders the transfer of electrons between the CB of Bi_2_S_3_ and that of CeO_2_. Additionally, this alteration also restricts the flow of holes, preventing them from moving from the VB of CeO_2_ to the VB of Bi_2_S_3_.^29,66^ Upon solar illumination, the holes and electrons generated by the excitation of CeO_2_ and Bi_2_S_3_, in the presence of IEF, the electrons in the CB of CeO_2_ are moved to the interface and recombine with the holes from the VB of Bi_2_S_3_, which are also transferred to the interface. By eliminating unwanted electrons and holes through recombination, holes on CeO_2_ VB and electrons on Bi_2_S_3_ CB are retained and accumulated, which are strongly oxidizing and reducing, respectively.^25^ And a more direct degradation of TC by holes on CeO_2_ VB. The CB of Bi_2_S_3_, measured at −0.49 eV, which is higher compared to the −0.33 eV of O_2_/·O_2_^−^ (*vs.* NHE). In this process, the electrons in the CB facilitate the reduction of dissolved O_2_ in water to produce ·O_2_^−^, which subsequently reacts with TC to contribute to its degradation. Specific reactions are represented by eqn (6)–(10).

6 CeO_2_ + *hv* → e^−^(CeO_2_) + h^+^(CeO_2_)

7 Bi_2_S_3_ + *hv* → e^−^(Bi_2_S_3_) + h^+^(Bi_2_S_3_)

8 h^+^(CeO_2_) + TC → products

9 e^−^(Bi_2_S_3_) + O_2_ → ·O_2_^−^

10 ·O_2_− + TC → products

**Fig. 11:**
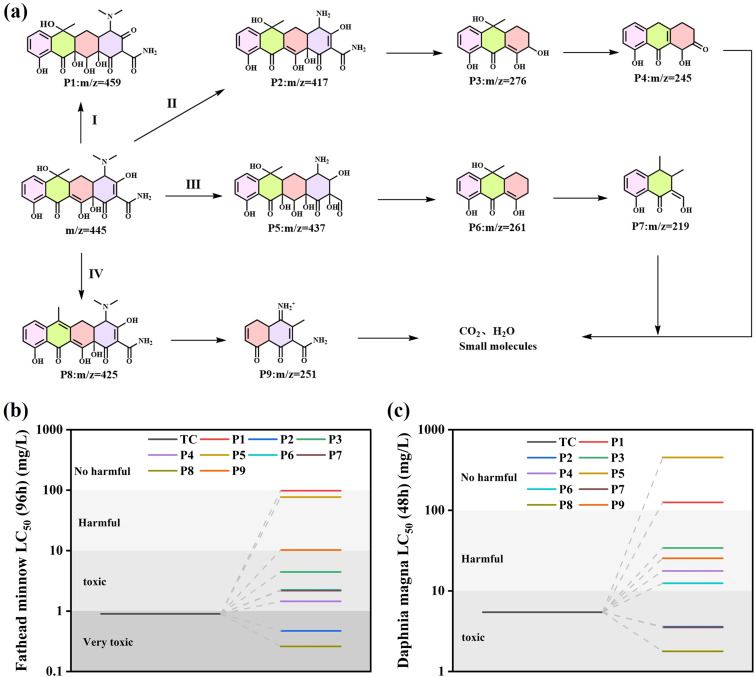
(a) Possible intermediates and pathways for TC degradation by CeO_2_/Bi_2_S_3_-2 photocatalysts; (b) fathead minnow LC_50_ (96 h); (c) *Daphnia magna* LC_50_ (48 h).

The intermediates of TC degradation by CeO_2_/Bi_2_S_3_-2 at different light durations were explored by mass spectrometry (MS). The peak that was recorded at an *m*/*z* value of 445 was linked to the original molecule TC. As the duration of light exposure increased, the intensity at *m*/*z* = 445 significantly diminished. In addition, peaks at *m*/*z* values of 459, 437, 425, 417, 276, 261, 251, 245 and 219 were identified, with the related MS spectra displayed in Fig. S1(a)–(f).† Based on the detected intermediates and available literature, a pathway for TC degradation was raised (Fig. 11(a)). In pathway I, the TC molecule undergoes a process known as hydroxylation, which results in the formation of a new compound designated as P1 (*m*/*z* = 459). In pathway II, the N–C bond's low binding energy makes it susceptible to destruction, leading to demethylation that produces P2 (*m*/*z* = 417). Subsequently, P2 experiences a ring-opening reaction, yielding P3 (*m*/*z* = 276), which further experiences dehydroxylation and dehydration to form P4 (*m*/*z* = 245). In pathway III, the TC molecule experiences several hydroxylation and demethylation processes to produce P5 (*m*/*z* = 437). This process subsequently entails a ring-opening reaction followed by dehydroxylation, resulting in the generation of a product designated as P6 (*m*/*z* = 261). Subsequently, P6 also undergoes ring-opening and dehydroxylation, resulting in the formation of P7 (*m*/*z* = 219). For pathway IV, the TC molecule dehydroxylates one hydroxyl group to produce P8 (*m*/*z* = 425), which in turn generates P9 (*m*/*z* = 251) by ring opening and demethylation and dehydroxylation. TC is broken down into intermediates with smaller molecular weights, which are further oxidized to small molecular compounds or even to CO_2_ and H_2_O.

In addition, the toxicity of TC and intermediates was evaluated using the Toxicity Estimation Software (T.E.S.T), and the specific values are shown in Table S2.† TC was evaluated to be highly toxic to fathead minnow (LC_50_ = 0.9 mg L^−1^) and toxic to *Daphnia magna* (LC_50_ = 5.44 mg L^−1^). The LC_50_ of all products except P8 was higher than that of TC (Fig. 11(b)), and the LC_50_ of most products was higher than that of TC for *Daphnia magna* (Fig. 11(c)), which indicated that CeO_2_/Bi_2_S_3_-2 degraded TC into a low toxicity compound.

## 4 Conclusions

In this study, flower-shaped CeO_2_/Bi_2_S_3_ composite photocatalysts were synthesized using hydrothermal methods. Characterization using SEM, TEM and XRD confirmed that the CeO_2_/Bi_2_S_3_ is a heterojunction. Through XPS analysis, DRS analysis, DFT calculation and Mott–Schottky tests, the CeO_2_/Bi_2_S_3_ heterojunction was found to function according to a charge transfer mechanism of the S-scheme type, which enhances charge mobility and generates an internal electric field that effectively separates electron–hole pairs. This process maintains the redox capacity of the resultant beneficial electrons and holes, thereby improving photocatalytic degradation efficiency. Under optimal reaction conditions, a significant degradation of TC was observed following 120 min of exposure to light. The results indicated that the degradation rate reached an impressive 82.43%. Free radical trapping experiments and LC-MS analysis of degradation products revealed that TC degradation primarily involves ·O_2_^−^ and h^+^ radicals, leading to the formation of CO_2_, H_2_O, and other small molecules through a series of ring-opening, demethylation, and dehydroxylation reactions. This research provides robust evidence for the photocatalytic breakdown of antibiotics through S-scheme heterojunctions and presents a viable approach for developing heterojunctions that integrate wide bandgap and narrow bandgap semiconductors.

## Author contributions

Shanlin He: writing – original draft, investigation, writing – review & editing, formal analysis, data curation. Yawei Du: formal analysis, visualization. Chen Li: resources, supervision. Claudia Li: writing – review & editing, supervision. Jingde Li: writing – review & editing, supervision. Jaka Sunarso: writing – review & editing, supervision. Sibudjing Kawi: writing – review & editing, supervision. Yinhui Li: writing – review & editing, supervision, project administration, funding acquisition.

## Conflicts of interest

There are no conflicts to declare.

## Data Availability

The data that has been used is confidencial.
